# Association between non-high-density lipoprotein cholesterol to high-density lipoprotein cholesterol ratio (NHHR) and sleep disorders in US adults: NHANES 2005 to 2016

**DOI:** 10.1097/MD.0000000000038748

**Published:** 2024-06-28

**Authors:** Yuzhou Cai, Zengkai Zhou, Yujian Zeng

**Affiliations:** a Department of Gastrointestinal Surgery, The First Affiliated Hospital of Kunming Medical University, Yunnan, China.

**Keywords:** cross-sectional study, NHANES, NHHR, sleep disorders

## Abstract

NHHR (non-high-density lipoprotein cholesterol to high-density lipoprotein cholesterol ratio) is a novel lipid parameter. However, the association between NHHR and sleep disorders remains unknown.; A cross-sectional analysis was conducted using data from the National Health and Nutrition Examination Survey (NHANES) 2005 to 2016. The association between NHHR and sleep disorders was explored using weighted multivariate logistic regression and generalized summation models. Subgroup analyses were employed to verify the robustness of this association. The prevalence of sleep disorders was 25.83% in a total of 22,221 participants. Compared to the lowest quartile of NHHR, participants in the top quartile had a 14% higher odds of sleep disorders prevalence in fully adjusted model (OR: 1.14, 95% CI: 1.06–1.23). After subgroup analyses and interaction tests, sex, race, marital status, education level, body mass index (BMI), person income ratio (PIR), alcohol consumption, smoking status, hypertension, and diabetes mellitus were not significantly associated with this positive association (P for interaction > 0.05). The NHHR is positively associated with sleep disorders in US adults. The management and monitoring of NHHR may have a potential role in improving sleep disorders.

## 1. Introduction

Sleep is critical for maintaining mental and physical health, as well as having a significant impact on metabolism and immunity.^[1]^ Between 50 and 70 million Americans are affected by sleep disorders.^[2]^ These sleep disorders are linked to non-communicable diseases including obesity, diabetes, cancer, and depression.^[3]^

Therefore, identifying new biomarkers to assess the prevalence of sleep disorders is crucial for improving prevention and treatment strategies. The ratio of non-high-density lipoprotein cholesterol (non-HDL-C) to high-density lipoprotein cholesterol (HDL-C), termed non-high-density lipoprotein cholesterol to high-density lipoprotein cholesterol ratio (NHHR), serves as a novel marker of atherosclerotic lipid profiles.^[4]^ A growing number of studies have demonstrated that the quality of sleep has a significant impact on the prevalence of subclinical atherosclerosis.^[5]^ Poor sleep quality in diabetic patients is linked to insulin resistance and hyperlipidemia.^[6]^ NHHR demonstrates improved effectiveness over traditional lipid parameters in assessing the risk associated with cardiovascular and cerebrovascular diseases^[7]^ and is significantly associated with the stability of carotid plaques.^[8]^ Moreover, NHHR has been proven in past studies to be a more reliable marker than traditional lipid parameters for identifying insulin resistance.^[9]^ An independent relationship exists between extended sleep durations and insulin resistance.^[10]^ Consequently, as a recently identified lipoprotein ratio, the NHHR merits further investigation in the context of sleep disorders.

Consequently, this study employed the National Health and Nutrition Examination Survey (NHANES) dataset spanning the years 2005 through 2016 to investigate the association between NHHR levels and sleep disorders among US adults.

## 2. Materials and methods

### 2.1. Study population

**Figure 1.**
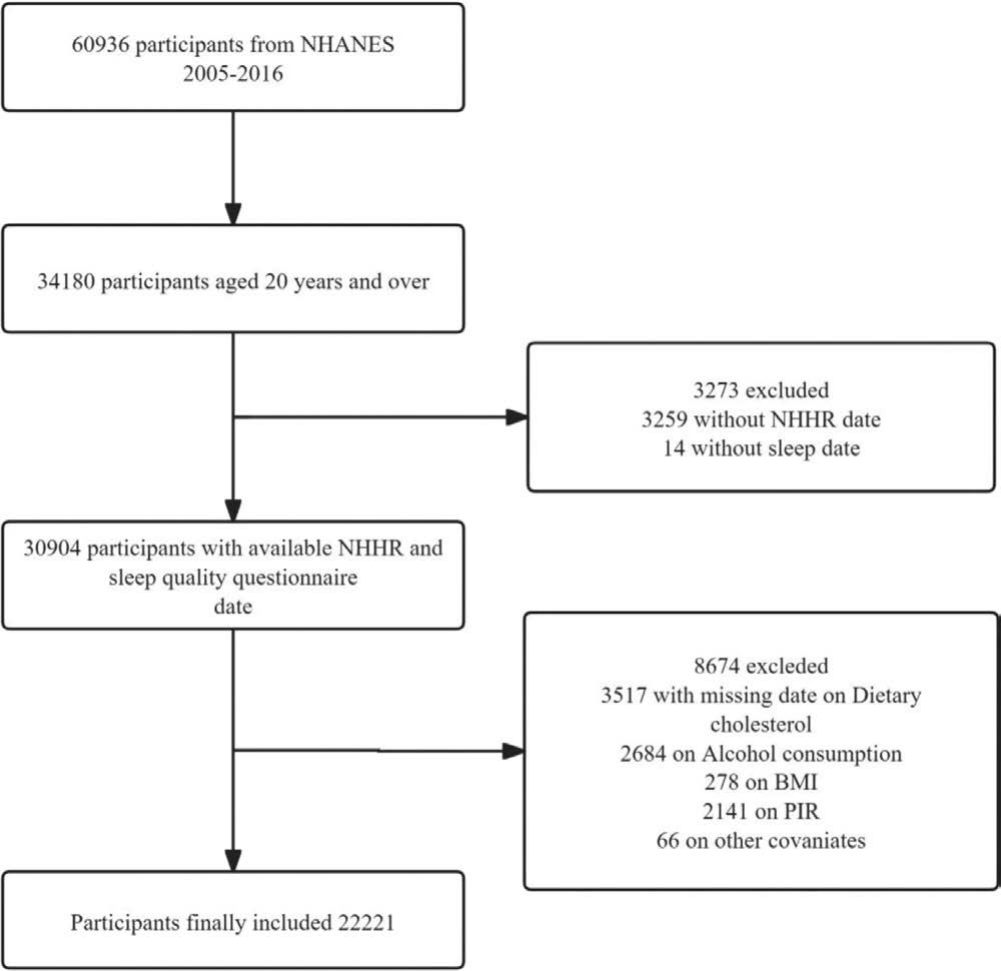
Flowchart of the population included in our study. BMI = body mass index, NHHR = non-high-density lipoprotein cholesterol to high-density lipoprotein cholesterol ratio, PIR = person income ratio.

This study analyzed data from NHANES from 2005 to 2016. Its data collection methodology combined structured interviews and physical examinations to collect comprehensive data on demographic characteristics, socioeconomic status, dietary patterns, and health-related issues. All study procedures were approved by the Ethics Review Board of the National Center for Health Statistics prior to data collection, and all participants signed informed consent forms. Visit https://www.cdc.gov/nchs/nhanes/index.htm for details on the process.^[11]^ The NHANES 2005 to 2016 cohort consisted of 60,936 individuals. In this study, we excluded individuals under 20 years of age, individuals with missing sleep data and missing NHHR data, and individuals missing data on key covariates; a detailed flowchart of this process is provided in Figure 1.

### 2.2. NHHR

The calculation formula for NHHR is: NHHR = Non-HDL-C/ HDL-C

### 2.3. Sleep disorders assessment

Use questions from the official NHANES website to screen for sleep disorders: “Have you ever reported to a doctor or other health professional that you have trouble sleeping?.” Participants who responded positively were classified as having a sleep disorder.

### 2.4. Covariates

The study a priori controlled for a range of potential confounding explanandum, including sociodemographic data, lifestyles, and health status factors. Age, sex, ethnicity, and educational attainment were included in the sociodemographic data. Marital status was dichotomized as married or unmarried, and income was stratified into 3 categories: <1.3, ≥1.3 and < 3.5, ≥3.5.

In this study, we conducted a comprehensive examination of lifestyle-related confounding variables. Smoking status was dichotomized into nonsmokers and regular smokers (defined as individuals who smoked 100 cigarettes or more). Alcohol consumption was dichotomized into nondrinkers and regular drinkers (defined as individuals who consumed 12 or more drinks per year). Dietary cholesterol intake was quantified through 2 24-hour dietary recalls, and the average intake was calculated based on detailed nutrient data.

Indicators of health status, such as body mass index (BMI), were measured at a mobile examination center (MEC). BMI was divided into 3 groups (BMI < 25; 25 ≤ BMI < 30; BMI ≥ 30). The study also assessed HDL cholesterol, diabetes mellitus, and hypertension. The history of diabetes was determined by a physician diagnosis or the use of insulin, whereas hypertension was determined by a self-reported diagnosis. The serum cholesterol testing was conducted in accordance with the protocol documented on the official NHANES website.

### 2.5. Statistical analysis

The sample weights from the official NHANES website were employed to ensure an accurate representation of the population. Continuous variables will be presented as means and standard deviations, while categorical variables will be expressed as percentages. Analyses employed both continuous and categorical models. To assess differences between groups, weighted Student t tests were employed based on the presence of sleep disorders, while weighted chi-square tests were utilized to analyze the relationship between groups. Furthermore, the relationship between NHHR and sleep disorders was investigated using multivariate logistic regression, which allowed for the examination of independent associations between the 2 variables.

Model 1 did not include any covariates. Contrastingly, Model 2 adjusted for demographics and health-related factors such as gender, age, ethnicity, marital status, educational attainment, BMI, and person income ratio (PIR). Model 3 extended these adjustments to incorporate smoking status, alcohol consumption, diabetes, hypertension, HDL-C, and dietary cholesterol. Non-linear relationships between NHHR and sleep disorders were analyzed using weighted Generalized Additive Models (GAM) for curve fitting and regression.

Subgroup analyses were conducted via stratified multivariable regression, stratifying by gender, age, ethnicity, BMI, PIR, level of education, marital status, hypertension, diabetes, alcohol use, and smoking status. The robustness of the model was verified by means of the log-likelihood ratio test.

All analyses utilized R software (version 4.3.2), available at http://www.R-project.org, with procedures implemented through EmpowerStats, accessible at www.empowerstats.com.

## 3. Results

### 3.1. Baseline characteristics of participants

A total of 22,221 participants were included in this study. Among them, 25.83% reported experiencing sleep problems. The weighted results indicate that the average participant age was 47.33 years, with males representing 48.26% of the cohort. The mean values of NHHR were 2.96 for participants without sleep disorders and 3.03 for those with sleep disorders, showing a significant difference (*P* < .05). The weighted baseline characteristics of the participants are shown in Table 1. The participants with sleep disorders were predominantly female, non-Hispanic white, married, smokers, and alcoholics. Additionally, these participants had greater BMI and PIR values, with all these differences reaching statistical significance (all *P* < .05).

**Table 1:** Weighted baseline characteristics of included participants.

Characteristic	Total	Without sleep disorders. (n = 16489)	With sleep disorders. (n = 5732)	*P* value
Age (yr)	47.33 ± 16.79	46.05 ± 17.05	50.76 ± 15.56	<.0001
Gender (%)				<.0001
Male	48.26	50.65	41.83	
Female	51.74	49.35	58.17	
Race (%)				<.0001
Mexican	8.09	9.29	4.89	
Other Hispanic	4.76	5.21	3.58	
Non-Hispanic White	70.27	67.68	77.24	
Non-Hispanic Black	10.28	10.65	9.27	
Other Race	6.59	7.17	5.02	
Education level (%)				<.0001
Less Than 9th	4.66	4.75	4.39	
9–11th	10.32	10.70	9.30	
High School	22.48	21.92	23.99	
Some College	32.03	31.21	34.25	
College Graduate	30.51	31.42	28.07	
Marital status (%)				<.0001
Unmarried	41.67	40.51	44.79	
Married	58.33	59.49	55.21	
Income-poverty ration				.0001
<1.3	20.66	19.98	22.51	
≥1.3, <3.5	35.30	35.81	33.94	
≥3.5	44.03	44.21	43.55	
BMI (kg/m^2^)				<.0001
<25	29.85	31.45	25.56	
≥25, <30	33.42	34.18	31.39	
≥30	36.73	34.38	43.05	
TC (mg/dL)	195.91 ± 41.50	195.31 ± 40.76	197.52 ± 43.38	.0004
HDL-C (mg/dL)	53.43 ± 16.41	53.37 ± 16.24	53.57 ± 16.87	.4190
Dietary cholesterol (mg)	288.39 ± 183.58	291.00 ± 184.29	281.37 ± 181.49	.0005
NHHR	2.98 ± 1.47	2.96 ± 1.41	3.03 ± 1.64	.0017
Diabetes (%)				<.0001
No	90.87	92.29	87.07	
Yes	9.13	7.71	12.93	
Alcohol consumption (%)				.0008
No	22.88	23.46	21.34	
Yes	77.12	76.54	78.66	
Hypertension (%)				<.0001
No	68.21	72.61	56.40	
Yes	31.79	27.39	43.60	
Smoking status (%)				<.0001
No	54.85	58.49	45.10	
Yes	45.15	41.51	54.90	

BMI *=* body mass index, HDL-C *=* high-density lipoprotein cholesterol, NHHR = non-high-density lipoprotein cholesterol to high-density lipoprotein cholesterol ratio, TC = Total cholesterol.

### 3.2. Association of NHHR and sleep disorders

**Figure 2.**
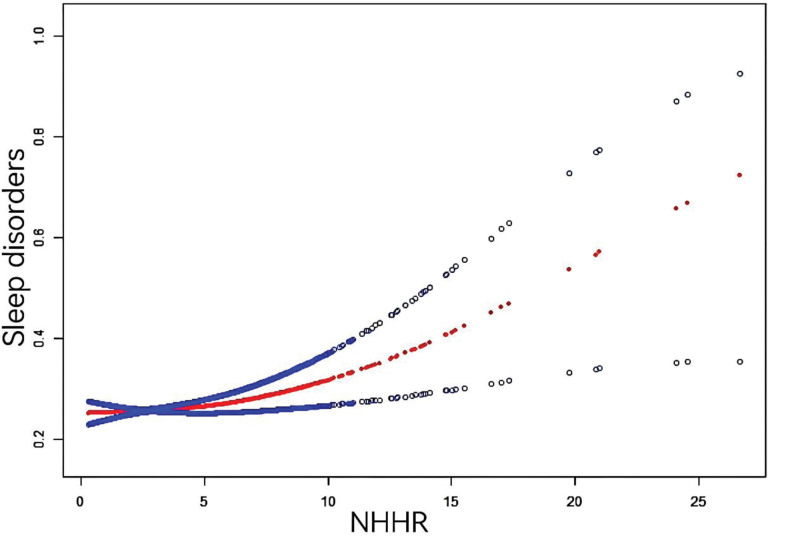
The association between NHHR and sleep disturbances. The solid red line represents the smooth curve fit between the variables. NHHR = non-high-density lipoprotein cholesterol to high-density lipoprotein cholesterol ratio.

Table 2 delineates the interrelationship between NHHR and sleep disorders. In the continuous model, after full adjustment for covariates, NHHR was found to be significantly associated with sleep disorders (OR 1.05, 95% CI: 1.01–1.09, *P* = .0186), with a 5% increase in the prevalence of sleep disorders. Significant associations were also observed in Model 1, which was not adjusted for variables (OR 1.03, 95% CI: 1.00–1.06, *P* = .0339), and in Model 2, which was adjusted for demographic variables (OR 1.07, 95% CI: 1.04–1.10, *P* = .0001). In Model 3 categorical model, odds ratios for the prevalence of sleep disorders in the 3 remaining quartiles were 0.79 (0.61–1.22), 1.03 (0.74–1.42), and 1.14 (1.06–1.23), with no significant trend differences observed (*P* > .05). However, significant trend differences were noted in Model 2 categorical model (*P* < .05). Additionally, results from the smooth curve fitting analysis demonstrated a positive correlation between NHHR and the prevalence of sleep disorders (Fig. 2).

**Table 2:** Association between NHHR and sleep disorders among US adults in NHANES 2005 to 2016.

Outcomes	Continuous models	*P* value	Categorical model				
	OR		Q1	Q2	Q3	Q4	P trend
Model 1	1.03 (1.00–1.06)	.0339	1 (Ref)	0.79 (0.53–1.17)	1.00 (0.73–1.32)	1.13 (1.06–1.21)	0.768
Model 2	1.07 (1.04–1.10)	.0001	1 (Ref)	0.83 (0.54–1.25)	1.02 (0.75–1.39)	1.15 (1.07–1.23)	0.009
Model 3	1.05 (1.01–1.09)	.0186	1 (Ref)	0.79 (0.61–1.22)	1.03 (0.74–1.42)	1.14 (1.06–1.23)	0.806

Model 1 did not adjust for potential confounders. Model 2 included adjustments for demographic variables such as sex, age, ethnicity, marital status, educational level, BMI, and PIR. Model 3, based on Model 2, further adjusted for factors such as smoking status, alcohol consumption, diabetes, hypertension, high-density lipoprotein cholesterol, and dietary cholesterol.

### 3.3. Subgroup analysis

**Figure 3.**
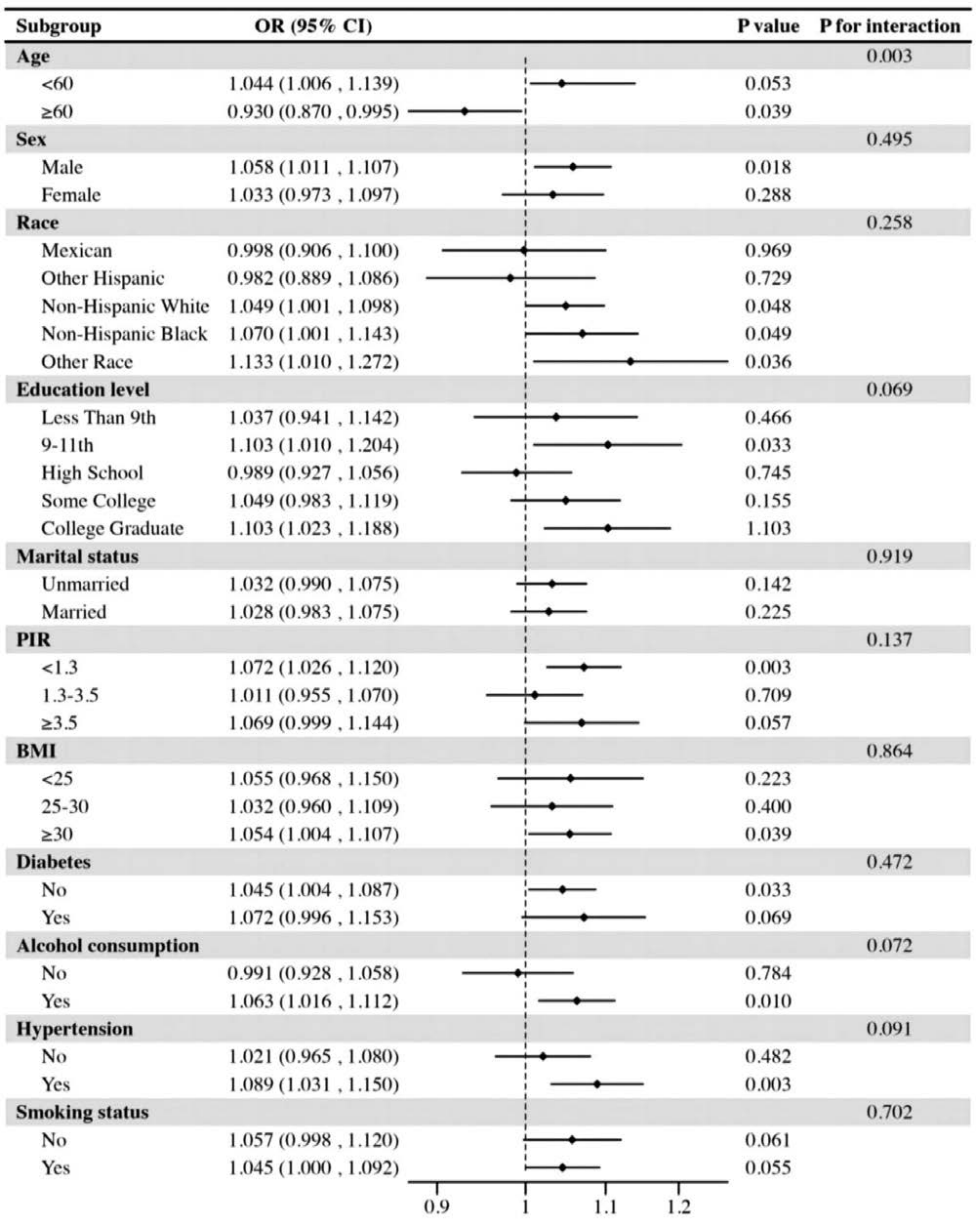
Subgroup analysis of association between NHHR and sleep disorders; BMI subgroup: BMI < 25; 25 ≤ BMI < 30; BMI ≥ 30; PIR subgroup: PIR < 1.3; 1.3 ≤ PIR < 3.5; PIR ≥ 3. BMI = body mass index, PIR = person income ratio.

Subgroup analyses revealed that the correlation between NHHR levels and sleep disorders was inconsistent (Fig. 3). Significant correlations between NHHR and sleep disturbances were observed in subgroups of sex, age, race, education level, BMI, PIR, hypertension, diabetes, and alcohol consumption (*P* < .05). Additionally, positive correlations between NHHR and sleep disorders were observed in subgroups divided by marital status and smoking status, although these were not statistically significant (*P* > .05). Interaction tests showed that the association between NHHR and sleep disorders was not statistically different across subgroups, indicating that variables such as gender, race, education, marital status, BMI, PIR, hypertension, diabetes mellitus, alcohol intake, and smoking habits did not significantly alter this association (*P* > .05 for interaction).Nevertheless, a noteworthy interaction was identified between age and NHHR, with the relationship exhibiting a statistically significant association (P for interaction < 0.05).

## 4. Discussion

We conducted a cross-sectional study to examine the potential association between the NHHR and the prevalence of sleep disorders in the US adult population. Our findings indicate that elevated levels of NHHR are associated with an increased prevalence of sleep disorders, after adjusting for sociodemographic factors, lifestyle choices, and health status. This apparent association was also consistent across multiple groups when stratified by age, PIR, BMI, education, presence of alcohol, history of hypertension, and history of diabetes.

NHHR represents a novel lipid ratio for evaluating atherosclerotic lipid profiles. An increasing amount of research indicates that NHHR is a more potent predictor of risk for diseases associated with lipids,^[12–14]^ including its superiority over traditional lipid parameters in assessing the extent of atherosclerosis.^[15]^ Although the role of NHHR in sleep disorders has not been previously studied, the connection between HDL-C and sleep quality has been extensively explored. A cross-sectional study comprising 60,283 participants demonstrated that alterations in serum lipid levels were associated with alterations in sleep duration. Lower HDL-C levels are significantly associated with shorter sleep duration in men.^[16]^ Similarly, another cross-sectional study identified low HDL levels as independent predictors of poor sleep quality.^[17]^ The results of a Mendelian randomized study indicate a correlation between elevated risks of developing sleep apnea and lower levels of HDL-C.^[18]^ These related studies’ conclusions may indirectly support our findings.

The utilization of novel lipid characteristics facilitates the expansion of the corpus of literature investigating the association between lipid profile and sleep disorders. A recent study demonstrated the superior predictive ability of the NHHR for nonalcoholic fatty liver disease (NAFLD).^[19]^ Furthermore, NHHR serves as an effective tool for assessing insulin resistance, offering greater predictive precision for diabetes-related conditions than conventional lipid tests.^[20]^ Recent studies also suggest significant associations between NHHR and depression symptoms,^[21]^ suicidal ideation,^[22]^ periodontitis,^[23]^ and kidney stones.^[24]^ Overall, NHHR demonstrates exceptional predictive efficacy in various studies. Moreover, NHHR noninvasive characteristics, ease of acquisition, and cost-effectiveness make it a method with significant potential for broad clinical application. Consequently, our research offers novel insights that facilitate a more comprehensive understanding of the relationship between HDL-C levels and sleep disorders. The results of our analysis indicated that the outcomes of the NHHR differed in the continuous and categorical models. Although the correlation appeared positive, these results should be interpreted cautiously. Subgroup analysis revealed a notable interaction between age and NHHR. A review noted that older people do not sleep as well as younger people.^[25]^ A cross-sectional study observed fluctuations in lipid levels with age and an association between age and non-HDL cholesterol levels in a group comprising individuals aged 57 years and older.^[26]^

Limited research has been conducted on the mechanisms linking lipid levels to sleep disorders. Lipids play an integral role in the maintenance of cell membrane structural integrity and functionality, including those in neurons involved in regulating sleep. Alterations in lipid composition can affect membrane fluidity and receptor function, thereby affecting sleep-related neurotransmitter signaling.^[27,28]^ Specific lipids can act as signaling molecules that modulate the activity of pathways involved in sleep-wake regulation, for instance, certain fatty acids can bind to receptors in the brain that affect sleep structure and stability.^[29,30]^ Obstructive sleep apnea, among the most frequent sleep disorders, is correlated with modified lipid profiles.^[31]^ A study has observed a correlation between obstructive sleep apnea and lower HDL cholesterol levels.^[32,33]^ In addition, metabolic disorders characterized by dyslipidemia, such as obesity and diabetes mellitus, have been associated with sleep disorders. This relationship may be mediated through mechanisms such as inflammation and oxidative stress, which are exacerbated by abnormal lipid metabolism and affect neuronal circuits controlling sleep.^[34,35]^ It has been demonstrated that alterations in inflammatory features also affect serum cholesterol.^[36]^ The inhibition of inflammatory cell activation by HDL-C has been demonstrated.,^[37]^ HDL-C reduces the concentration of various inflammatory cytokines, including TNF-α, and has the function of promoting the production of endothelial-type nitric oxide synthase (eNOS).^[38]^ Apolipoprotein A-I accounts for approximately 70% of the HDL protein composition, while apolipoprotein A-II accounts for approximately 20%.^[39]^ Apolipoprotein A-I is crucial for activating lecithin-cholesterol acyltransferase (LCAT), exhibiting both anti-inflammatory and antioxidant properties.^[40,41]^ In addition, HDL-C exerts antioxidant effects by reducing lipid peroxidation and maintaining mitochondrial energy production.^[42]^ However, lower HDL-C levels in patients with sleep disorders impair these protective effects. These insights into the relationship between lipid metabolism and sleep highlight potential therapeutic targets for improving sleep health by modulating lipid pathways. Our research demonstrates a significant association between NHHR and a high prevalence of sleep disorders. Furthermore, the study also highlights the negative effects of low HDL-C levels, which warrant further investigation to elucidate the direct mechanisms involved.

The use of a large, nationally representative sample of US adults represents a significant strength of this study. Consequently, we have conducted a comprehensive adjustment for a range of variables, thereby enhancing the reliability and validity of our findings. It should be noted that our study is not without limitations. Specifically, the cross-sectional design inhibits our ability to establish causality between NHHR and sleep disorders, raising the possibility of reverse causation. Considering the complexity of factors affecting sleep, we included as many covariates as possible. Secondly, our analysis relied solely on fasting cholesterol measurements, without considering non-fasting data. Differences in laboratory test results may lead to bias. Third, we collected data on sleep disorders using the NHANES-designed questionnaire, which constrains further exploration of the connection between NHHR and the severity of sleep disorders. To the greatest extent feasible, our large population sample was able to compensate for some deficiencies. Fourth, while we accounted for several potential covariates, fully eliminating confounding factors after adjustment remains challenging within the confines of our study. Fifth, while our analysis identified variations in the relationship between NHHR and sleep disorders among various income and racial groups, more future research is needed to explore how economic and cultural factors influence sleep health through biological pathways.

## 5. Conclusions

Our findings suggest that NHHR levels are significantly associated with the prevalence of sleep disorders in US adults. However, this association requires further prospective studies and randomized controlled trials, as well as further research into the mechanisms underlying this association. In light of these findings, future public health interventions for sleep health and related health problems, as well as individualized treatment and prevention strategies, should consider NHHR levels to minimize associated risks.

## Acknowledgments

We thank the staff at the National Center for Health Statistics of the Centers for Disease Control for designing, collecting, and collating the NHANES data and creating the public database.

## Author contributions

**Conceptualization:** Yuzhou Cai.

**Data curation:** Yuzhou Cai.

**Formal analysis:** Yuzhou Cai.

**Funding acquisition:** Yuzhou Cai.

**Investigation:** Yuzhou Cai.

**Methodology:** Yuzhou Cai.

**Project administration:** Yuzhou Cai.

**Resources:** Yuzhou Cai.

**Software:** Yuzhou Cai.

**Supervision:** Yuzhou Cai, Yujian Zeng.

**Validation:** Yuzhou Cai.

**Visualization:** Yuzhou Cai.

**Writing – original draft:** Yuzhou Cai, Zengkai Zhou.

**Writing – review & editing:** Yuzhou Cai.
